# 1220. Comparing Breakthrough Fungal Infections in Acute Myeloid Leukemia (AML) and Myelodysplastic Syndrome (MDS) Patients Receiving Prophylaxis with Different Azoles

**DOI:** 10.1093/ofid/ofad500.1060

**Published:** 2023-11-27

**Authors:** Catherine Chen, Frank P Tverdek, Zahra Escobar, Catherine Liu, William F Simmons, Hanh Bui

**Affiliations:** UW Medicine, Seattle, Washington; Seattle Cancer Care Alliance, Seattle, Washington; University of Washington, Seattle, Washington; Fred Hutchinson Cancer Research Center, Seattle, Washington; University of Washington, Seattle, Washington; UW Medicine/Fred Hutchinson Cancer Center, Seattle, Washington

## Abstract

**Background:**

Posaconazole is recommended for primary prophylaxis in neutropenic patients with acute myeloid leukemia (AML) or myelodysplastic syndrome (MDS) receiving induction chemotherapy. Despite prophylaxis, breakthrough invasive fungal infections (bIFIs) still arise. Initiating posaconazole is not always feasible, and may necessitate use of alternative azoles. The primary objective of this study was to evaluate the rate of bIFIs in patients receiving prophylaxis with posaconazole versus alternative azoles. The secondary objective reviewed reasons for alternative azole use.

**Methods:**

A retrospective chart review was conducted at University of Washington Medical Center (UWMC) and Fred Hutchinson Cancer Center (FHCC). Patients who received induction chemotherapy for AML/MDS from March 2021 to November 2022 and received a prophylactic azole for at least 7 days during neutropenia were included. Outcomes were evaluated with Chi-Square. The incidence of bIFI was evaluated up to 12 weeks from induction chemotherapy, or until consolidation chemotherapy or hematopoietic stem cell transplant, whichever came first.

**Figure:**
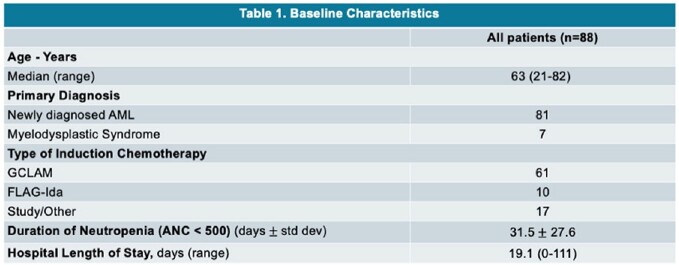

**Results:**

The initial query yielded 175 patients with AML/MDS. Of these, 88 patients were included: 74 received posaconazole and 14 received an alternate azole (Table 1). BIFIs occurred in 9 patients (posaconazole (n=7, 9.5%), alternative azole (n=2, voriconazole, 14.2%); p=0.631) (Figure 1). The most common reason for initiating an alternative azole was insurance restrictions/high cost (Figure 2).

**Figure:**
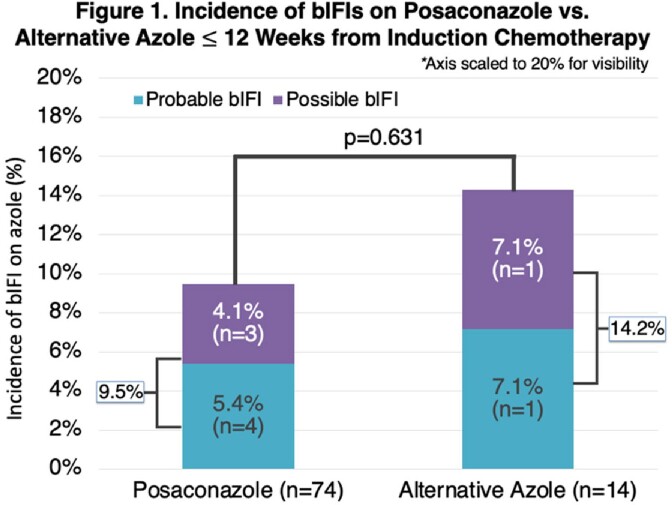

**Figure:**
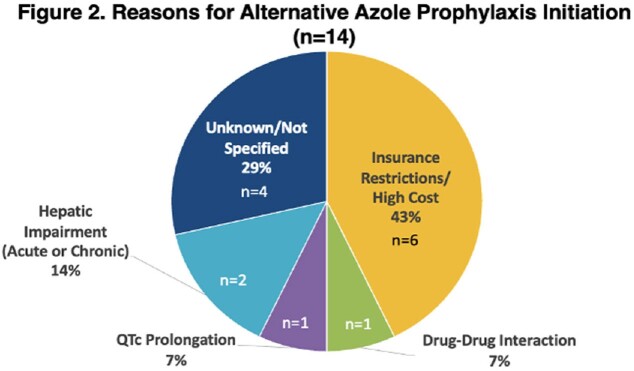

**Conclusion:**

Numerically, we found reduced incidence of bIFIs among patients receiving posaconazole compared to alternative azoles. Insurance restrictions/cost was the most common reason for alternative azole utilization. This barrier may increase risk of bIFIs, and additional efforts should be considered to use posaconazole over alternative azoles.

**Disclosures:**

**Catherine Liu, MD**, Pfizer: Site Investigator|SNIPR BIOME: Advisor/Consultant

